# Effect of Cholesterol Removal Processing Using ***β***-Cyclodextrin on Main Components of Milk

**DOI:** 10.1155/2013/215305

**Published:** 2013-06-25

**Authors:** A. M. Maskooki, S. H. R. Beheshti, S. Valibeigi, J. Feizi

**Affiliations:** ^1^Food Processing Department, Research Institute of Food Science and Technology (RIFST), Km. 12 Asian Road, Mashhad, Iran; ^2^TESTA Quality Control Laboratory, North-East Food Technology and Biotechnology Zone, Km. 12 Asian Road, Mashhad, Iran

## Abstract

Various concentrations (0%, 0.5%, 1% and 1.5%) of *β*-CD were mixed with different fat contents (1%, 2.5% and 3%) of raw (unhomogenized) and homogenized milk at two mixing temperatures of 8 and 20°C. The cholesterol residue, fat, protein, lactose, solid nonfat (SNF), density, and ash content of milk were measured for each treatment. The results statistically analysed and showed that the cholesterol content of milk remarkably decreased as the *β*-CD was increased particularly in homogenized milk at 20°C. However, the reduction rate of cholesterol was decreased when extra *β*-CD was added due to its intermolecular reactions. The maximum cholesterol reduction was achieved at the level of 1% *β*-CD. The fat content, SNF, protein, lactose, and density content were decreased with increasing *β*-CD whereas it did not affect ash content.

## 1. Introduction

According to the World Health Organization, 2010 report, cardiovascular diseases are the first among the top 10 causes of death. An estimated 16.7 million people or 29.2% of total global deaths are due to the various forms of cardiovascular disease (CVD) [1, 2]. The direct relation between high blood cholesterol and CVD has been proved. Cholesterol is a typical animal sterol; for example, its content in milk fat is 95%–98% [3]. The main source of cholesterol in food comes from animal origin, such as meat, milk, and eggs. The other major source of cholesterol is produced by the liver in the body [4]. Milk and dairy products contain relatively high level cholesterol that can elevate the blood cholesterol [5]. First attempts on reducing cholesterol in food go back to early twentieth century. Denis and Minot [6] determined the cholesterol content of animal and human milks and they suggested the relation between blood plasma cholesterol and food intake. Although so many papers were published about the relation of food intake and increasing of blood plasma cholesterol, no serious attempt took place on reducing cholesterol in food until 1960. Since the 1960s, large number of physicochemical methods was recommended to reduce cholesterol in food as well as blood cholesterol [7]. Cholesterol could be removed in an efficient manner from milk fat up to 90% using supercritical CO_2_ technology [8]. Bobby and Joseph Jr. [9] developed and patented a process for the production of cholesterol-free milk based on extraction of cholesterol from the milk fat globule membranes using an organic polar solvent and without substantial loss of solid milk fat. Solvent-free and low cholesterol products were recovered from the reseparated and washed cream fractions. Cholesterol removal from dairy product using saponin and diatomaceous earth also was patented by Richardson et al. [10]. They suggested that the process could be useful particularly for raw and pasteurized milks, cream, and butter. In spite of effectiveness of the mentioned methods, some complications exist because of the organic solvents and saponin residues in food and safety problems for human body. The worries about solvent residue and harmful saponin consumption for human health motivated the investigator to find nontoxic and effective substances instead of unsafe materials. In the last years, several studies describing the use of beta cyclodextrin (*β*-CD) and food applications have been published [11]. It has been proved that the *β*-CD molecule can be used as nontoxic and nondigestible molecule to remove cholesterol effectively from milk and animal products for improving their nutritional characteristics [12]. *β*-CD is a cyclic oligosaccharide consisting of seven glucopyranose molecules that are linked together with *α* 1–4 bonds. *β*-CD is frequently used as building blocks. More than 20 substituents have been linked to *β*-CD by a regioselective manner [13]. The most widespread use of *β*-CD is in cholesterol removal from animal products, such as eggs and dairy products. *β*-CD treated materials show 80% removal of cholesterol in various food containing cholesterol [14]. Chiu et al. [15] removed more than 85% cholesterol content of egg yolk by immobilization of *β*-CD on chitosan beads. The process for preparing low cholesterol dairy product using *β*-CD has been patented by Graille et al. [16]. Lee et al. [17] suggested that the 94.3% of cholesterol content of homogenized milk with 3.6% fat could be removed by addition of 1.5% *β*-CD at mixing temperature of 10°C for 10 min of mixing time. The method for removing cholesterol from milk and cream was developed and patented by Kwak et al. [18]. Optimization of cholesterol removal in cream using *β*-CD by response surface method was investigated by Ahn and Kwak [19]. Kim and coworkers [20–22] developed a cross-linked *β*-CD and epichlorohydrin to recycle the cholesterol removal process. They concluded that cross-linked *β*-CD would result in almost 100% effective recycling efficiency. Further, this method was applied and optimized in order to remove cholesterol from milk and dairy product and recover the cross-linked *β*-CD by Kwak et al. [23]. Recently, Dias et al. [24] investigated butter cholesterol removal using different combinatorial methods with *β*-CD. Although many investigations were carried out to demonstrate the feasibility of *β*-CD as excellent substance for removing cholesterol from food including milk, few investigations have been reported on the effect of *β*-CD on physicochemical properties of milk with various fat content. Ha et al. [25] measured the amount of nutrients (lactose, short-chain FFA, FAA, and water-soluble vitamins) that were entrapped during cholesterol removal from cream by cross-linked *β*-CD. They concluded that the amount of entrapped nutrients was negligible during cholesterol removal from cream by cross-linked *β*-CD. In spite of this study, the cholesterol removal process by *β*-CD may affect main components and physicochemical properties of milk. The aims of this study are evaluation of the feasibility of *β*-CD in cholesterol removal from raw and homogenized milks with various fat contents and the effect of cholesterol reduction process on main components of milk.

## 2. Materials and Methods

### 2.1. Raw Materials and Reagents

Commercial raw (unhomogenized) and homogenized milks with 1%, 2.5%, and 3% fat content were purchased from a Pegah Dairy Co. Industry as needed (located in Mashhad Iran).

*β*-CD, cholesterol, and 5*α*-cholestane were purchased from Sigma Chemical Co. (St. Louis, MO, USA). All reagents and solvents were gas-chromatographic grade.

### 2.2. Cholesterol Removal Processing

Overall, 100 mL of raw or homogenized milk with various fat contents (1%, 2.5%, and 3%) were separately placed in 500 mL beakers and different concentrations of *β*-CD (0%, 0.5%, 1%, and 1.5%) were added to each of samples. The mixture was stirred vigorously by VELP stirrer 700 rpm at two temperature conditions of 8 and 20°C for 10 and 15 min. The mixtures were centrifuged with Eppendorf centrifuge model 5810 at 112–448 ×g for 15 min. The supernatant containing cholesterol reduced milk was decanted and removed for further measurements. The protein, lactose, fat, solid nonfat (SNF), ash, and density were measured using MilkoScan device MCC model (Milkotronic CO., Bulgaria).

### 2.3. Modified Cholesterol Analysis

The determination of cholesterol was carried out using HPLC-SYKAM, S1122 (Germany) with capillary column Symmetry C_18_, length of 25 cm, diameter 4.6, vol. 5 *μ*m, and UV detector 205 nm. The temperature of column was adjusted to 40°C using column oven S4011. The preparation of solutions and reagents method was described by Klatt [26]. We have used this method with some modifications. Accurately 30 mL milk with constant fat content was poured in 250 mL Erlenmeyer flask. Also, 40 mL of 95% ethanol and 8 mL of 50% KOH (W/W) were added to flask. Then, the flask was placed on a magnetic heater-stirrer, connected to condenser, and stirred vigorously for 70 min to ensure the saponification reaction was completed. Once more, 60 mL of 95% ethanol were added and stirring procedure was continued without heating. The condenser was disconnected and the flask was removed after 15 min. The saponified solution flask was topped with stopper and rested to be cooled at room temperature for 24 h. The extraction procedure was carried out by adding 100 mL toluene to saponified sample while stirring for 30 min. The solution was poured into 500 mL decantation funnel without rinsing and then 110 mL of KOH were added. The decantation funnel was shaked up vigorously for 10 sec. We let layers be separated and then we discarded the aqueous lower layer. The extraction stage was repeated by adding 40 mL of 0.5 N KOH solutions. The aqueous solution was discarded and the toluene was washed with 40 mL H_2_O for three times. The toluene residue was evaporated by rotary vacuum evaporator. The remained cholesterol was dissolved in 5 mL methanol HPLC grade and was injected to HPLC device.

### 2.4. Determination of Main Components of Milk

The determination of main components and some physicochemical properties of milk was carried out by ultrasonic milk analysis device.  Table 1 shows the capability and accuracy of ultrasonic milk analysis.

### 2.5. Statistical Procedure

Each experiment was repeated at least three times. Multifactor Randomized Complete Block Design (RCBD) was used to analyse the acquired raw data statistically. The obtained means were evaluated by Duncan Multiple Range Test (D.M.R.T). Statistical analysis was performed using Sigma Stat 3.1, MiniTab15, and Microsoft Excel softwares.

## 3. Results and Discussions

### 3.1. Effect of *β*-CD Concentration

**Figure 1:**
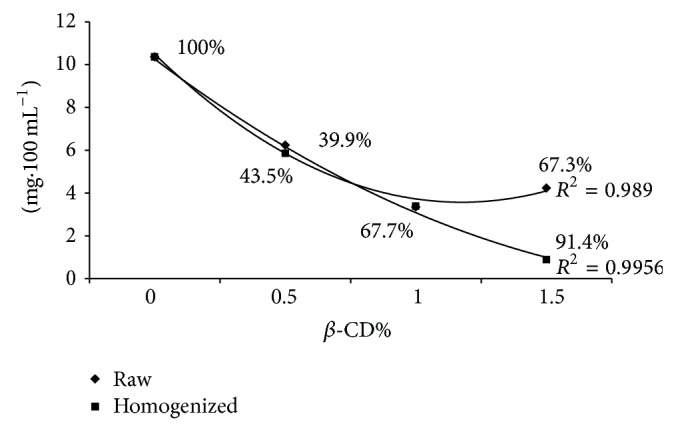
Cholesterol reduction in both raw and homogenized milks by various amount of *β*-CD.

The cholesterol content of both raw and homogenized milks was significantly (*P* ≤ 0.05) reduced with increasing the *β*-CD.  Figure 1 shows the cholesterol residues and percentage reduction of cholesterol in treated raw and homogenized milks. The reduction effect is more obvious in homogenized milk than in the raw one. However, there were no remarkable differences between the reduction rates of raw and homogenized milks at 0.5% and 1% *β*-CD concentrations (*P* ≥ 0.05). The absorption of cholesterol was reduced when using 1.5% *β*-CD particularly in raw milk. The obtained results in this study showed that cholesterol is effectively removed from milk and dairy product by *β*-CD which is in agreement with other investigators [16, 17, 27, 28]. The lipid complexes in homogenized milk are significantly smaller than unhomogenized (raw), and therefore the possibility of cholesterol molecule inclusion by internal cavity of *β*-CD is higher in homogenized milk than in raw one. The cholesterol removal trend was slightly decreased when 1.5% *β*-CD was applied particularly in raw milk. This phenomenon can be due to intermolecular reaction effects of extra *β*-CD. Kim et al. (1999) have suggested that an excess of *β*-CD could compete with itself to bind to cholesterol molecules, and consequently cholesterol adsorption is decreased. Therefore, it seems that the concentration of 1% *β*-CD may be sufficiently effective to remove greater than 90% of cholesterol from homogenized milk as shown in Figure 1.

### 3.2. Effect of Operation Temperature

**Figure 2:**
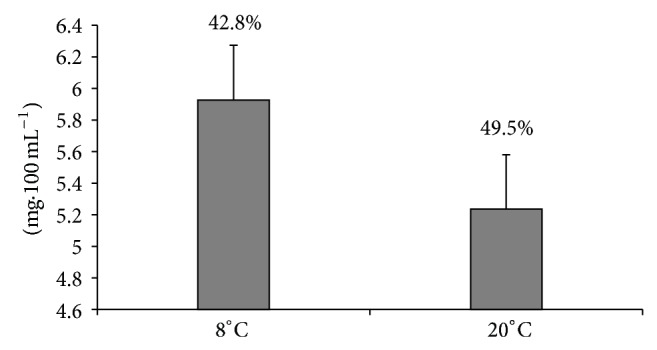
The cholesterol reduction in milk for both mixing temperature conditions.

The *β*-CD was added and mixed with milk samples at two temperatures 8 and 20°C for 15 min. The adsorption of cholesterol was significantly increased when *β*-CD was added and mixed at 20°C.  Figure 2 shows the effect of mixing temperature on reduction of cholesterol in treated milks. The differences were more obvious in higher concentration of *β*-CD. However, the diversity of opinions exists among investigators on this result. Lee et al. [17] reported that no difference was found in cholesterol removal at mixing temperature of 4, 10, 15, or 20°C, while Oakenfull and Sihdu [29] disagreed with this result. They indicated that the removal of cholesterol using *β*-CD significantly is influenced by temperature. They reported that 77%, 63%, and 62% cholesterol were removed when milk was treated with 1.0% *β*-CD at 4, 8, and 40°C, respectively, during 10 min of mixing. Yen and Tsui [30], and Kim et al. [22] also confirmed that the removal of cholesterol with *β*-CD from lard which was stirred at 50°C is greater in comparison with the temperature condition of 27 or 40°C. Actually, our overall results showed that the higher cholesterol removed when mixing temperature was increased as demonstrated in Figure 2. This result is in agreement with investigations concerning the effect of mixing temperature on the reduction of cholesterol by *β*-CD that were carried out by other investigators [22, 29, 30]. Naturally, the aggregations of milk lipoproteins at the mixing temperature of 20°C are lower than 8°C due to reaching of lipids to their melting points, and consequently there is more chance for the entrapment of molecules in *β*-CD cavities. Therefore, we can expect more cholesterol molecules to be trapped in *β*-CD cavities at higher temperatures. 

**Figure 3:**
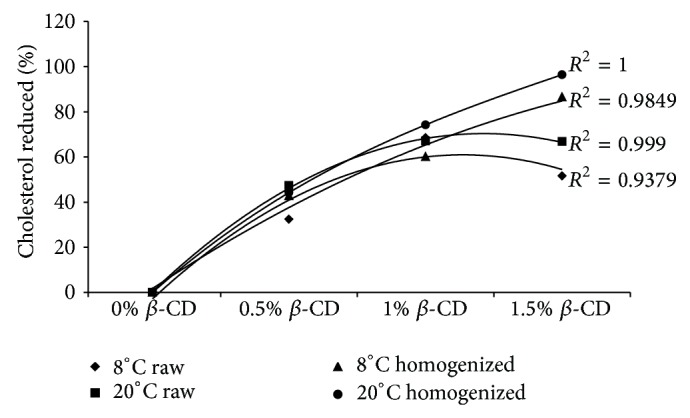
The cholesterol reduction in both raw and homogenized milks using various concentrations of *β*-CD for both temperature conditions.

**Figure 4:**
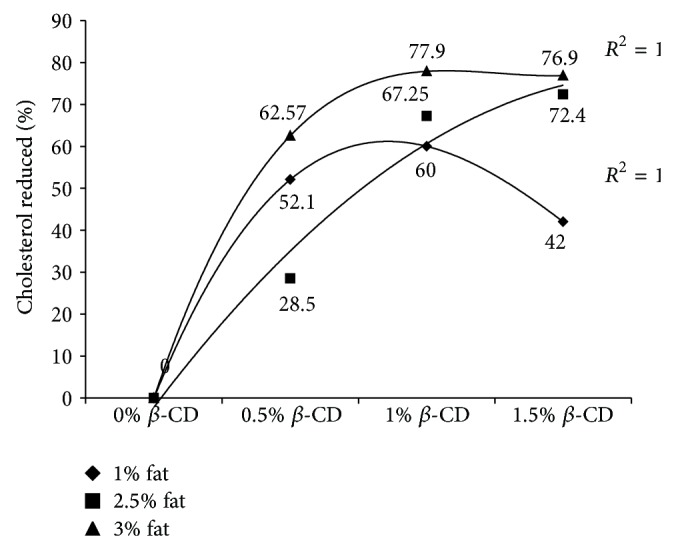
Cholesterol reduction (percentage) by various concentrations of *β*-CD in milk with different fat contents.

Figure 3 shows the amount of cholesterol reduction in both raw and homogenized milks in various concentrations of *β*-CD at both temperature conditions of 8 and 20°C. We observed that the increase of cholesterol removal depends on amount of *β*-CD in higher temperatures. There were no remarkable statistical differences between cholesterol reduction in both raw and homogenized milk samples at two mixing temperatures of 8 and 20°C treated with low concentrations of 0.5% and 1% of *β*-CD while the differences were significant when we applied higher concentration of 1.5% *β*-CD as shown in Figure 4. Kim et al. [22] confirmed this result by investigation on cholesterol reduction of lard using high concentration of *β*-CD.

### 3.3. Interaction Effects of Fat Content and Concentration of *β*-CD

More concentration of *β*-CD was needed with increasing the fat content in milk. The percentage of cholesterol, which was reduced by various concentrations of *β*-CD in different milk fat contents, is shown in Figure 4. The highest cholesterol reduction was achieved by 1% *β*-CD followed by 1.5% and 0.5% in 3% fat content of milk. The entrapped cholesterol molecules in *β*-CD cavities were reduced when we applied excess *β*-CD for milk samples with low fat content. The percentage of cholesterol reduction was only 42% when we applied 1.5% *β*-CD, while we achieved 52.1% and 62% with 0.5% and 1% *β*-CD, respectively. The best results were achieved when we applied 1% *β*-CD in various fat contents. The cholesterol reductions by 1% *β*-CD were 60%, 67.25%, and 77.9% for 1%, 2.5% and 3% of milk fat, respectively. The molecules of *β*-CD are linked together, therefore, the ability of cholesterol absorption is reduced. Dias et al. [24], Ahn and Kwak [19], and Kim et al. [22] have confirmed our results.

### 3.4. Effect of Cholesterol Reduction by *β*-CD on Physicochemical Properties of Milk

#### 3.4.1. Effect on Fat Content

The cholesterol removal processing by *β*-CD on total fat content of milk was investigated as shown in Table 2. Obviously, the fat content of milk was reduced when cholesterol was removed by *β*-CD because cholesterol molecules are a part of milk lipids. This reduction of fat in homogenized milk samples was more obvious than in raw milk. The fat content of homogenized milk was significantly decreased with increasing the amount of *β*-CD due to smaller size of fat globules in homogenized milk compared with raw milk. The trend of fat decrease in raw milk samples was slower than homogenized with increasing the concentration of *β*-CD. Aggregation of fat molecules in raw milk inhibits the inclusion of cholesterol in *β*-CD molecules. Ha et al. [25, 31] suggested that the free fatty acids in food may be reduced because they are trapped in *β*-CD molecule cavities. Kwak et al. [32] reported that cheddar cheese fat content made by milk treated by *β*-CD was lower than control milk samples. The cholesterol reduction processing such as stirring, separation, and centrifugation during separation of “*β*-CD + cholesterol” may affect fat reduction particularly in homogenized milk.

#### 3.4.2. Effect on Milk Protein

The milk protein content was decreased with increasing the cholesterol absorption by *β*-CD. The amount of protein was significantly decreased in high concentrations of *β*-CD particularly in homogenized, milk, and there is a direct relation between increasing the concentration of *β*-CD and decreasing the protein content of milk as shown in Table 2. Kwak et al. [32] and Kim et al. [21] have already reported the protein reduction in milk after removal of cholesterol. Suzuki [33] suggested that the bitter taste due to protein hydrolyzate can be removed by 10% *β*-CD. The protein molecules and protein-lipid networks are smaller in homogenized milk than in raw milk. Ha et al. [25] confirmed that the protein content of cream was reduced during reduction of cholesterol by 4%–10% *β*-CD due to entrapment of amino acids in *β*-CD cavities. On the other hand, the outer surface of *β*-CD macromolecules has affinity to absorb the negative charges that cover the protein surfaces. Consequently, parts of protein together with “*β*-CD + cholesterol” leave the environment during separation of *β*-CD. The cholesterol reduction processing such as mixing, separation, and centrifugation may affect decrease of milk protein.

#### 3.4.3. Effect on Nonfat Solid Content

The nonfat solid content of both raw and homogenized milks was slightly decreased after cholesterol reduction processing. Although cholesterol molecules are lipids and are independent to SNF, but presence of *β*-CD in milk may influence constituents such as proteins, ions, and lactose. Moreover, cholesterol removal operations may have effect on the reduction of nonfat solid content as shown in Table 2. The SNF was reduced with increasing the amounts of *β*-CD, and it was proved that some constituents of milk may entrapped in the cavity of *β*-CD and leave the environment after separation of *β*-CD. Ha et al. [31] confirmed the decreasing of some nutritional materials during cholesterol removal processing of cream using *β*-CD.

#### 3.4.4. Effect on Lactose

The same effect was observed on lactose. The lactose content in both raw and homogenized milks was decreased after cholesterol removal processing. This phenomenon was more obvious when the amount of *β*-CD increased particularly in homogenized milk as illustrated in Table 2. Ha et al. [25, 31] reported the effect of cholesterol removal using *β*-CD on lactose previously, and they noted that the reduction of lactose is very low and negligible. However, we guess that the decrease of lactose is due to cholesterol reduction processing and the ability to bind lactose to *β*-CD and subsequently exit the milk suspension in association with “*β*-CD + cholesterol” complex.

#### 3.4.5. Effect on Density

The density of milk strongly depends on existing of material in milk and is generally decreased when the main constituents of milk are reduced. The density of milk was significantly decreased after removing the cholesterol by *β*-CD because of losing some components of milk after cholesterol removal treatment. Decrease of milk density in homogenized milk is more than raw milk as illustrated in Table 2. The density of homogenized milk samples was significantly changed from 1.0313 (control) to 1.028 (treated samples) when 1.5% *β*-CD was applied to remove the cholesterol of milk. We could not find any published report about this phenomenon. 

#### 3.4.6. Effect on Ash Content

The effect of cholesterol minimization treating by *β*-CD had no remarkable effect on ash content of treated milk samples. Generally, the minerals of milk are sodium, potassium, calcium, and magnesium. These minerals could not be affected by *β*-CD as well as cholesterol removal operation and there was no remarkable effect on ash content of milk.

## 4. Conclusions

This study depicts the ability of various concentrations of *β*-CD to remove cholesterol from both raw and homogenized fresh milks and their effects on main constituents of milk. Many investigations have been carried out on cholesterol removal using *β*-CD. Undoubtedly, the ability of *β*-CD to exclusively remove cholesterol from milk has been proved and we confirmed these results. However, a few studies reported the effects of cholesterol reduction on nutritional compounds such as protein, lactose, and fat contents of milk. Although the *β*-CD molecules are edible and nontoxic and as a results they can be used safely as cholesterol removal agent from milk and dairy products, the effects of cholesterol removal processing are very important. This operations decreased the fat, density, SNF, and lactose content of milk. This phenomenon may be due to cohesiveness of these compounds with *β*-CD or existence some excess operations such as filtration and centrifugation during separation of “*β*-CD + cholesterol.” Further investigation needs to demonstrate the quality of milk after cholesterol removal processing and to develop prevention methods of leaving nutritional materials of milk in association with *β*-CD.

## Highlights

- The ability of *β*-CD to remove cholesterol from milk has been proved.
- The *β*-CD molecules are safe and can be used as cholesterol removal agent.
- The effects of cholesterol removal processing are very important.
- The cholesterol removal operations can be decreased some main components of milk.

## Figures and Tables

**Table 1:** Capability and accuracy of milk by ultrasonic milk analysis for determination of physicochemical properties of milk.

Parameter	Measuring range	Accuracy
Fat	0,01%–45%	±0,06%
Solids nonfat (SNF)	3%–40%	±0,15%
Density	1000–1160 kg/m	±0,3
Protein	2%–15%	±0,15%
Lactose	0,01%–20%	±0,2%
Added water	0%–70%	±3
Milk sample temperature	5°C–40°C	±1%
Freezing point	(−0,4°C)–(−0,7°C)	±0,005%
Salts	0,4%–4%	±0,05%
pH	0–14	±0,05%
Conductivity	2–14 (mS·cm^−1^)	±0,05%

**Table 2:** Effect of various concentrations of *β*-CD on main constituents and density of raw and homogenized milk.

Milk	*β*-CD (%)	Fat (g/100 g) SEM	Protein (g/100 g) SEM	SNF (g/100 g) SEM	Lactose (g/100 g) SEM	Density (g/100 mL) SEM	Ash (g/100 g) SEM
Raw	0	3.133 ± 0,002	3.1633 ± 0,011	8.633 ± 0.019	4.751 ± 0.08	1.0313 ± 0.003	0.714 ± 0.008
	0.5	3.111 ± 0,002	3.1025 ± 0,015	8.564 ± 0.03	4.733 ± 0.06	1.0311 ± 0.003	0.711 ± 0.008
	1	3.0908 ± 0,02	3.075 ± 0,016	8.535 ± 0.03	4.724 ± 0.05	1.0309 ± 0.003	0.711 ± 0.011
	1.5	3.0663 ± 0,003	2.9696 ± 0,014	8.424 ± 0.04	4.711 ± 0.02	1.0306 ± 0.004	0.713 ± 0.013

Homogenized	0	3.131 ± 0,002	3.1633 ± 0,011	8.633 ± 0.019	4.751 ± 0.08	1.0313 ± 0.003	0.714 ± 0.008
	0.5	2.993 ± 0,001	3.0575 ± 0,016	8.436 ± 0.04	4.662 ± 0.04	1.0285 ± 0.003	0.709 ± 0.008
	1	2.882 ± 0,003	3.0271 ± 0,015	8.406 ± 0.04	4.643 ± 0.04	1.0283 ± 0.007	0.712 ± 0.011
	1.5	2.805 ± 0,006	2.9475 ± 0,014	8.319 ± 0.05	4.642 ± 0.08	1.0280 ± 0.003	0.713 ± 0.013
